# Rheological Evaluation of Ultra-High-Performance Concrete as a Rehabilitation Alternative for Pavement Overlays

**DOI:** 10.3390/ma18153700

**Published:** 2025-08-06

**Authors:** Hermes Vacca, Yezid A. Alvarado, Daniel M. Ruiz, Andres M. Nuñez

**Affiliations:** 1 Department of Civil Engineering, Pontificia Universidad Javeriana, Carrera 7 No. 40-62, Bogotá 110231, Colombia; alvarado.y@javeriana.edu.co; 2 Bieton, Carrera 42#29-44, Itagüí, Medellín 055412, Colombia; proyectos@bietonco.com

**Keywords:** UHPFRC, rheology, pavement rehabilitation, yield stress, viscosity

## Abstract

This study evaluates the rheological behavior and mechanical performance of Ultra-High-Performance Fiber-Reinforced Concrete (UHPFRC) mixes with varying superplasticizer dosages, aiming to optimize their use in pavement rehabilitation overlays on sloped surfaces. A reference self-compacting UHPFRC mix was modified by reducing the superplasticizer-to-binder ratio in incremental steps, and the resulting mixes were assessed through rheometry, mini-Slump, and Abrams cone tests. Key rheological parameters—static and dynamic yield stress, plastic viscosity, and thixotropy—were determined using the modified Bingham model. The results showed that reducing superplasticizer content increased yield stress and viscosity, enhancing thixotropic behavior while maintaining ultra-high compressive (≥130 MPa) and flexural strength (≥20 MPa) at 28 days. A predictive model was validated to estimate the critical yield stress needed for overlays on slopes. Among the evaluated formulations, the SP-2 mix met the stability and performance criteria and was successfully tested in a prototype overlay, demonstrating its viability for field application. This research confirms the potential of rheology-tailored UHPFRC as a high-performance solution for durable and stable pavement overlays in demanding geometric conditions.

## 1. Introduction

The performance of fresh-state hydraulic concrete is a fundamental aspect of construction as it significantly influences a wide range of essential processes, including mixing, transportation, placement, compaction, finishing, and curing. These processes must be carefully controlled to meet the specific technical requirements of each project. Workability, a key property of concrete, is most commonly evaluated using the Slump test [1]. However, despite its widespread use, this test does not always provide a complete or accurate assessment of concrete’s workability. Two concrete mixtures with the same measured Slump value can exhibit vastly different flow characteristics, leading to significant variations in their performance. For instance, one mixture may demonstrate no flow at all (zero Slump), while another may exhibit high fluidity, effectively behaving as a self-leveling material, depending on its intended application. This limitation highlights the fact that the Slump test alone is insufficient to fully characterize the wide spectrum of workability properties in concrete [2,3,4]. To address this issue, the mini-Slump test [5] has been widely adopted for self-compacting concrete, offering a more reliable method for estimating workability and quantitatively determining the rheological properties of ultra-high-performance concrete (UHPFRC) [4,6,7]. Rheological properties provide a more comprehensive understanding of workability by defining critical physical parameters within rheological models. These models incorporate fundamental concepts such as thixotropy, which describes the time-dependent variation in shear stress when a material is subjected to a constant shear rate [8]. This behavior plays a crucial role in determining the flowability and stability of UHPFRC mixtures, influencing their overall performance in construction applications.

YS Tai et al. [9] highlighted that although it is widely recognized that achieving an optimal level of mix fluidity—neither excessively sticky nor overly fluid—is essential for ensuring the viability of concrete in construction applications, this characteristic is influenced by a range of factors that have yet to be fully explored in depth. These factors include the specific types of materials used in the mix, the mixing procedures employed, the properties and configurations of the mixing equipment, and external environmental conditions such as temperature, among others. Over the past few decades, extensive research has been conducted on novel cementitious materials aimed at overcoming the inherent limitations of conventional concrete. Several researchers [7,10,11,12,13] have contributed to this field by publishing studies on the development and optimization of Ultra-High-Performance Concrete (UHPFRC) and Fiber-Reinforced Ultra-High-Performance Concrete (UHPFRC), positioning these materials as key advancements in contemporary civil engineering.

UHPFRC is characterized by a dense, homogeneous, and self-compacting matrix, which is achieved through the precise combination of cementitious binders, fine aggregates, high-performance water-reducing additives, metallic or organic fibers, and a typically low water-to-binder (*w*/*b*) ratio, ranging from 0.14 to 0.20 [11]. Numerous studies have demonstrated that this advanced composite material exhibits superior mechanical performance and enhanced durability compared to conventional concrete [14,15,16]. In particular, various authors [17,18,19,20] have reported UHPFRC formulations achieving compressive strengths exceeding 150 MPa, further validating its potential as a high-performance material for structural applications. Due to these exceptional properties, the Federal Highway Administration [14] has documented an average compressive strength of 150 MPa for UHPFRC, along with a tensile strength exceeding 5 MPa, both before and after crack initiation.

The outstanding mechanical and durability properties of UHPFRC present significant opportunities for its application in the construction of new infrastructure as well as in the rehabilitation of existing pavements and bridges. These attributes contribute to the development of structures with enhanced longevity and improved structural performance [14,21,22,23]. Additionally, one of the key advantages of using UHPFRC in pavement rehabilitation is the potential for significantly reducing roadway closure times. The rapid strength gain observed at early ages enables the reopening of rehabilitated roadways in a considerably shorter timeframe compared to conventional concrete materials [24]. However, despite these advantages, the high fluidity and self-compacting nature of UHPFRC impose certain limitations when applied in pavement construction. Specifically, its flow behavior presents challenges in roadway installation, particularly in relation to design slopes, topographic constraints, environmental factors, and safety requirements [25].

Therefore, the implementation of UHPFRC in pavement applications requires a thorough evaluation and optimization of its on-site placement techniques in the fresh state. This necessitates a fundamental shift from the conventional high-fluidity rheological properties of UHPFRC towards a mix with thixotropic behavior—allowing the material to maintain its ultra-high-performance characteristics while improving its suitability for use in sloped pavement overlays. In this context, the present research focuses on evaluating the rheological behavior of UHPFRC mixes by systematically reducing the dosage of superplasticizer relative to a reference UHPFRC formulation. This adjustment aims to enhance the material’s thixotropic properties, thereby facilitating its application in pavement overlays with a 10% slope while preserving its exceptional mechanical performance and durability.

Based on the context described above, the novelty of the present research lies in evaluating the rheological behavior of thixotropic UHPFRC mixes specifically designed for pavement overlays on inclined surfaces. Unlike conventional self-compacting UHPFRCs, this study investigates how reducing the amount of superplasticizer affects workability, thixotropy, and mechanical performance. By correlating conventional tests, such as the mini-Slump with rheological parameters, and validating a critical yield stress model, the study proposes a practical method to select and apply UHPFRC mixes suitable for construction on slopes.

## 2. Materials and Experimental Design

The experimental program focused on analyzing key rheological parameters, specifically yield stress and viscosity, along with the overall fluidity of the reference UHPFRC mix. Additionally, these properties were evaluated in samples where the binder-to-superplasticizer ratio was systematically varied, allowing for an assessment of how these parameters evolved over time. Furthermore, a comprehensive characterization of the material in its hardened state was conducted for each evaluated mix proportion to better understand the impact of these variations on mechanical performance.

### Materials

The formulation of the different UHPFRC mixes incorporated type III high early-strength Portland cement [26], which is known for its rapid strength gain. Additionally, silica fume, calcium carbonate, and siliceous sand with a maximum particle size of 600 µm were used as supplementary components to enhance material performance. A polycarboxylate-based superplasticizer (SP), compliant with ASTM C494 [27] standards for type A (water-reducing) and type F (high-range water-reducing) admixtures, was employed to improve workability and optimize the rheological properties of the fresh mix (Table 1). For fiber reinforcement, steel fibers with a nominal diameter of 0.20 mm ± 0.006 mm were incorporated into the mix. These fibers exhibited a length-to-diameter aspect ratio of 65, contributing to improved mechanical properties, including enhanced tensile strength, ductility, and crack resistance. The selection of these materials aimed to achieve an optimized balance between workability, mechanical performance, and durability, ensuring that the UHPFRC mix met the stringent requirements for its intended applications.

Four (4) UHPFRC mixes with varying proportions of superplasticizer were produced, as detailed in Table 2. The SP-0 mix, designated as the reference mix, featured a self-compacting composition with a superplasticizer-to-binder ratio (SP/b) of 0.0196. This formulation was used as the baseline for comparison against modified mixes with reduced superplasticizer content, aiming to analyze the impact of incremental reductions on the rheological properties and workability of the material.

The other three mixes—SP-1, SP-2, and SP-3—were prepared with progressively lower SP/b ratios of 0.0188, 0.0180, and 0.0172, respectively. These reductions corresponded to a decrease in superplasticizer dosage relative to the reference mix: 4% for SP-1, 8% for SP-2, and 12% for SP-3 (Table 2). Previous trials indicated that reductions beyond 12% led to a significant loss of workability in the UHPFRC, while reductions smaller than 4% produced negligible changes in rheological behavior. The water-to-binder ratio (*w*/*b*) remained constant at 0.17 across all mixes to ensure that variations in fluidity and rheological behavior were solely influenced by changes in superplasticizer content. Likewise, the proportions of the remaining materials—Portland cement, silica fume, calcium carbonate, siliceous sand, and steel fibers—remained unchanged for all mixes.

The primary objective of this variation in the SP/b ratio was to evaluate how decreasing the superplasticizer dosage affected the yield stress, viscosity, and thixotropic behavior of fresh UHPFRC while also assessing potential implications for mechanical performance in the hardened state. By maintaining the same solid constituents and adjusting only the superplasticizer content, this study aimed to systematically quantify the impact of reduced fluidity on mix stability, workability, and applicability in pavement overlays.

The manufacturing of UHPFRC mixes was carried out using a planetary mixer with a total capacity of 0.1 m^3^. The mixing procedure for each batch followed a structured and controlled sequence, as detailed in Table 3, ensuring consistency in material preparation. The entire process, from the initial combination of components to the final homogenization, was completed within an average time of 31 min, corresponding to Steps 1 through 9 in Table 3. The procedure began with the mixing of superplasticizer and water (Step 1), followed by the incremental addition of dry materials in three stages (Steps 2, 4, and 6), ensuring uniform dispersion. Each addition was followed by a mixing phase (Steps 3, 5, and 7) to promote homogeneous integration of the materials. This resulted in a total mixing time of 27 min, after which steel fibers were incorporated (Step 8). The final homogenization stage (Steps 9–10) ensured even fiber distribution within the mix. Following the mixing process, testing in the fresh state was conducted for each mix at predefined time intervals: 40 min (t_40), 60 min (t_60), 80 min (t_80), 100 min (t_100), and 120 min (t_120). These evaluations, labeled as Simultaneous Tests in Table 3, included the following:

- Measurement of rheological parameters using a rheometer, determining key properties such as yield stress and viscosity.
- A mini-Slump test [5] was carried out, and laying was carried out with an Abrams cone [1], for each of the mixes SP-0, SP-1, SP-2, and SP-3, for each of the times defined, as depicted on Table 3.
- Evaluation of flow behavior using the Abrams cone test [1] to analyze consistency and laying characteristics.

This systematic testing methodology provided a comprehensive assessment of the rheological evolution and workability of the mixes, ensuring a robust understanding of how time-dependent variations influence UHPFRC performance in its fresh state.

**Figure 1:**
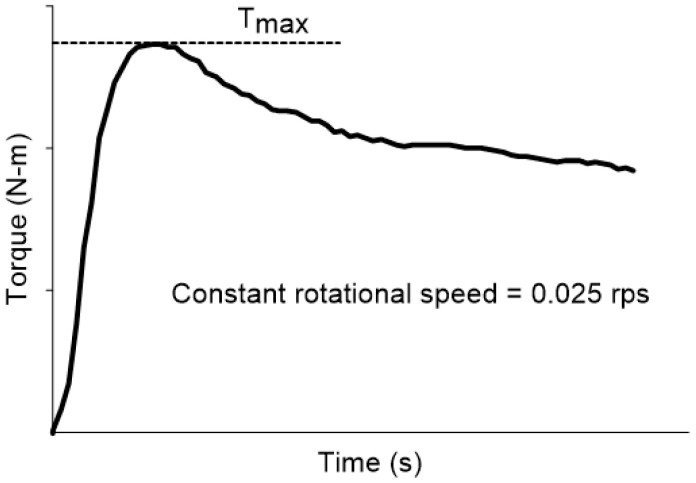
Maximum torque measurement method.

An ICAR vane rheometer equipped with four blades was utilized in this study to measure torque at different rotation speeds, enabling the determination of key rheological parameters for each UHPFRC mix. These parameters included the static yield stress ($\tau_{s}$), which represents the initial resistance to flow, as well as the flow stress ($\tau_{0}$) and plastic viscosity ($\mu_{p}$), both of which are essential for characterizing the material’s fresh-state behavior. The vane, connected to the rheometer, featured a diameter-to-height ratio specifically designed to optimize measurement accuracy. The device was submerged in a cylindrical container with an internal diameter of 260 mm and a height of 400 mm, ensuring a standardized testing environment. Once the mix was fully prepared, it was carefully poured into the container, and the rheometer was installed, following a strict protocol to minimize inconsistencies in testing conditions. This entire setup process was completed within an average time of 3 min, as outlined in Step 11 of Table 3. To determine the static yield stress ($\tau_{s}$), torque measurements were recorded as a function of time at a constant and very low rotational speed of 0.025 rms. This slow rotation allowed for the precise capture of the material’s elastic response before reaching the peak stress value, after which the material transitioned into a plastic flow state. The time–torque graph (Figure 1) illustrates this behavior, highlighting the elastic deformation phase leading up to the peak, followed by the plastic flow phase, where the material begins to yield under applied stress [27,28,29,30]. The static yield stress ($\tau_{s}$) was calculated using the following equation (Equation (1)):

(1) $$\tau_{s}=(2\cdot T_{max})/(\pi \cdot D^{3}\cdot (H/D+1/3))$$

where $\tau_{s}$ represents the static yield stress, $T_{max}$ is the maximum torque, D is the vane diameter, and H is the vane height. In this equation, it is assumed that shear stress is uniformly distributed along both the lateral surface and the ends of the vane. Consequently, the shear stress calculated for the concrete mix increases linearly as shear deformation progresses. Once the internal structure of the mix is disrupted, leading to the solid-to-liquid transition, the applied stress begins to decrease. At this point, the maximum recorded stress value corresponds to $\tau_{s}$, representing the material’s resistance to initial flow before yielding occurs [31].

**Figure 2:**
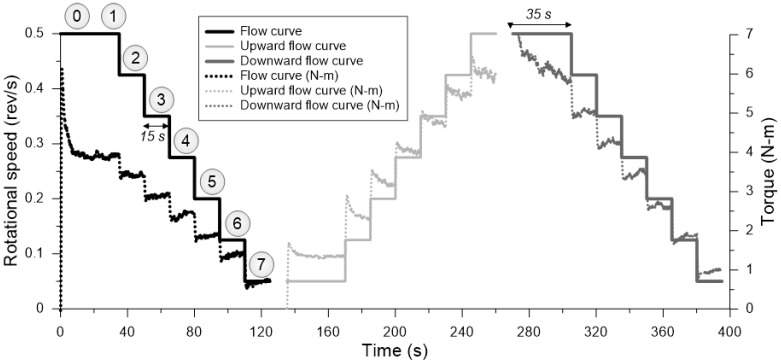
Method for the determination of rheological parameters and thixotropy in the evaluated mixtures.

To determine yield stress and viscosity, measurements were conducted immediately after recording $\tau_{s}$. These rheological parameters were obtained through a sequence of three standardized test procedures. The first test involved the measurement of the flow curve, which was assessed using eight (8) sequential steps over a total duration of 125 s (Figure 2). The initial step (Step 0) corresponded to a breakdown phase, during which the impeller speed (N) was set to 0.5 rps for 20 s. This preliminary phase was designed to mitigate thixotropic effects by disrupting the internal structure of the material before further rheological measurements were conducted [27]. Following this, the impeller speed was gradually reduced in incremental steps (0.50, 0.425, 0.35, 0.025, 0.200, 0.125, and 0.050 rps) to facilitate the recording of torque (T) values at each speed level. Each measurement step lasted 15 s, ensuring sufficient time for structural breakdown and stabilization of torque values. The torque–rotational velocity data obtained from the descending flow curve exhibited a linear relationship, consistent with the behavior of a Bingham fluid, as described by the Bingham model [32]. The corresponding rheological parameters were determined using the mathematical formulation represented in Equation (Equation (2)).

(2) T=Y+V·N

where Y (expressed in N·m) represents the intercept of the torque versus rotational speed curve, and V (in N·m/s) corresponds to the slope of the linear fit. The recorded torque and rotational velocity values were subsequently converted into fundamental rheological parameters, specifically yield stress ($\tau_{0}$) and plastic viscosity ($\mu_{p}$), using the Reiner–Riwlin equations, as defined in Equations (3) and (4) [33].

(3) $$\tau_{0}=Y/(4\cdot \pi \cdot h)(1/({R1}^{2})-1/({R2}^{2}))-1/ln(R2/R1)$$

(4) $$\mu =V/(8\cdot \pi^{2}\cdot h)(1/({R1}^{2})-1/({R2}^{2}))$$

where h is vane height (m), R1 is vane radius, and R2 is container radius. Rheological units were analyzed with the Bingham model (Equation (5)), with Y being shear deformation and T applied yield stress. However, other studies [34,35,36,37] have evaluated the rheology of self-compacting UHPFRC concrete using the modified Bingham model. This is because applied T, in some cases, presents a negative value. Equation (6) represents the modified Bingham model, with C being the second-order parameter. In this case, model adjustment always resulted in correlation coefficient values ($R^{2}$) greater than 0.93.

(5) $$\tau =\tau_{0}+\mu_{p}\cdot \gamma^{\cdot }$$

(6) $$\tau =\tau_{0}+\mu_{p}\cdot \gamma^{\cdot }+C\cdot ({\gamma^{\cdot }}^{2})$$

**Figure 3:**
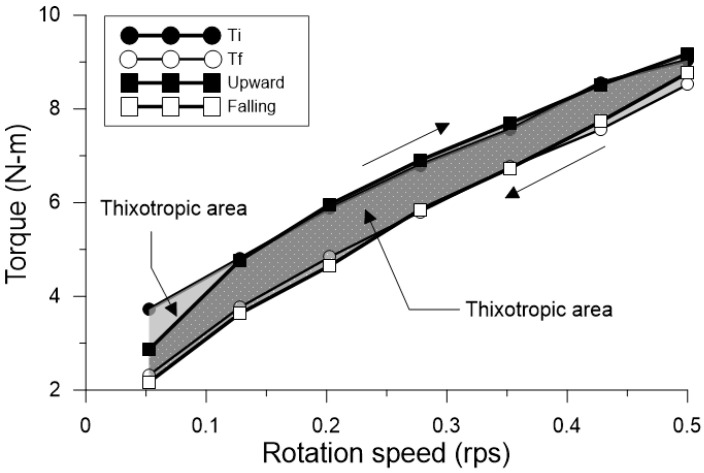
Measuring thixotropy using the Ahari et al. [39] model, and the ascending and descending shear ramp method, for SP-2 mix in t_120.

In the second sequence, an ascending flow ramp was made, with velocity of eight (8) steps, using the same velocity values described in the first sequence. Finally, the third sequence, was identical to the first sequence. The second and third sequences were used to quantify thixotropy, with measuring defined as the area between ascending and descending flow curves (Figure 3). Thixotropy of cement-based materials is typically measured in yield stress with relation to the variation of time [38].

With data obtained from the second test sequence, a method proposed by Ahari et al. [39] was adapted for the times described in this methodology. Therefore, the initial torque (Ti) was obtained from the average of data recorded in the first and second test, in the referenced velocity, and for each of the eight (8) sequences indicated in Figure 3. Final torque (Tf) was obtained from the average of data recorded in the last second of the 15 test seconds, or the equilibrium zone. The area between the initial torque curve and the final torque was used to quantify thixotropy, as observed in Figure 3, for times of 40 (t_40), 60 (t_60), 80 (t_80), 100 (t_100), and 120 min (t_120), as indicated in Table 3, Steps 12 to 16.

For characterization in a hardened state, cylinder and prism-shaped samples were manufactured between the times t_60 and t_80 mentioned in Table 3 for the mechanical characterization, through compressive resistance tests ($\sigma_{cs}$) [40] and flexural resistance [41]. Samples were evaluated at ages of 7, 14, and 28 days. Density values of samples were measured, whereby for every age, five (5) samples were assayed reporting standard deviation. Samples were cured in conditions of 23 °C and a relative humidity greater than 95%.

**Figure 4:**
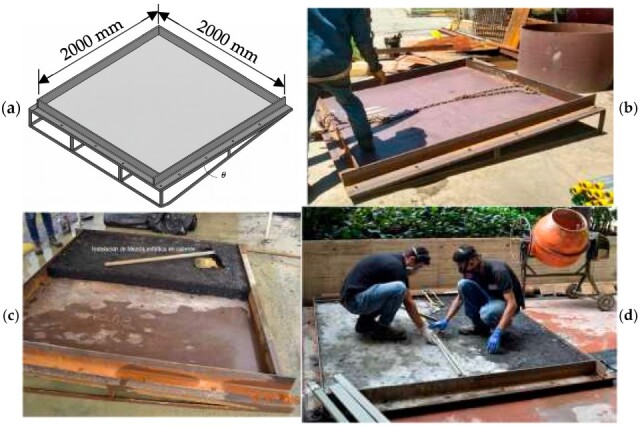
(**a**) Test formwork; (**b**) preparing of supporting layer; (**c**) UHPFRC support layer in asphaltic mix; (**d**) UHPFRC layer in conventional concrete.

Additionally, and according to previous studies reported in references [25,42], an experimental overlay-installing model can be achieved by taking a bi-dimensional flat deformation geometry independently to the longitudinal or transversal slope. Therefore, in this research, the model proposed was designed for installing a UHPFRC square overlay of 2000 mm × 2000 mm, with 40 mm in thickness, in a slope of 10% (Figure 4). Thickness was defined according to design recommendations for ultra-thin Whitetopping slabs [43]. The placed mix was restrained in its lower face by two types of materials that are usually used in pavement construction: the first one, a conventional concrete layer with a flexural resistance of 4 MPa; the second, an asphaltic mix with a resilient modulus of 3500 Mpa, Figure 4c and Figure 4d, respectively.

Out of the evaluated mixes SP-0, SP-1, SP-2, and SP-3, one was selected in order to verify mix quality to be used as an alternative in the installing of overlays, under criteria that encompassed mechanical performance, workability, and rheological parameters. The criterion for UHPFRC overlay stability or critical yield stress applied in this study was the one proposed by Tae Yong and others [25], defined as

(7) $$\tau_{c}=\lambda \cdot \rho \cdot g\cdot d\cdot tan\theta$$

where ρ is the UHPFRC density (kg/m^3^), g is the gravitational acceleration (9.81 m/s^2^), λ is the non-negative coefficient that describes the border condition, and d corresponds to the overlay thickness.

## 3. Results and Discussion

### 3.1. Characterization in Fresh State

**Figure 5:**
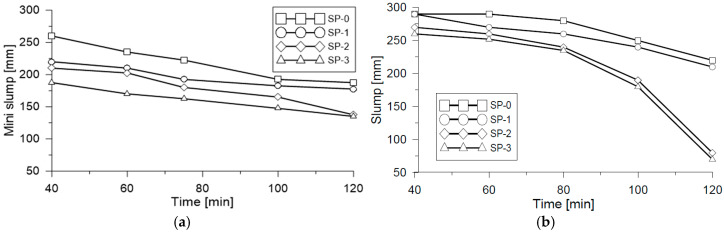
Mix fluidity measuring. (**a**) Mini-Slump test; (**b**) laying measuring using the Abrams cone.

**Figure 6:**
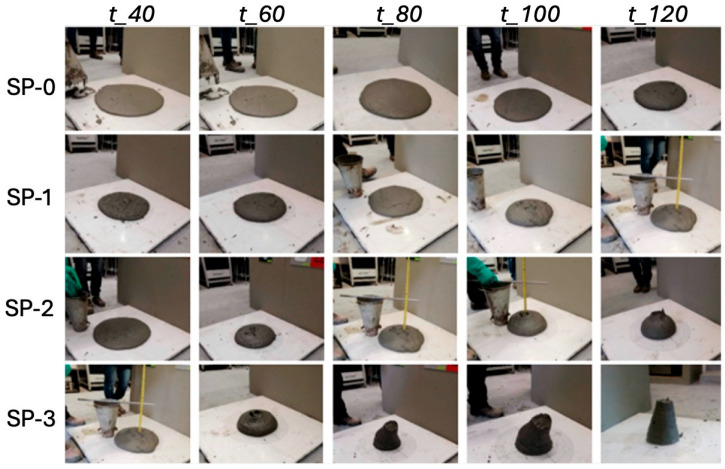
Measuring of Slump laying for study mixes.

For the evaluation of fluidity, the mini-Slump test [5] flow results and Abrams cone [1] for the different mixes in the study are depicted in Figure 5a and Figure 5b, respectively. In both tests, the flow dropped progressively with the reduction in the proportion of superplasticizer. This is attributed to electrostatic and/or steric repulsion effects with the cementing material molecules. Understanding that SP efficacy depends on the adsorption of superplasticizer molecules in the cement particles and the repulsion force developed by the adsorbed molecules [44,45]. A graphical summary of the Slump is presented in Figure 6, illustrating change in the material’s fluidity for SP-2 and SP-3 mixes in the analyzed time interval.

**Figure 7:**
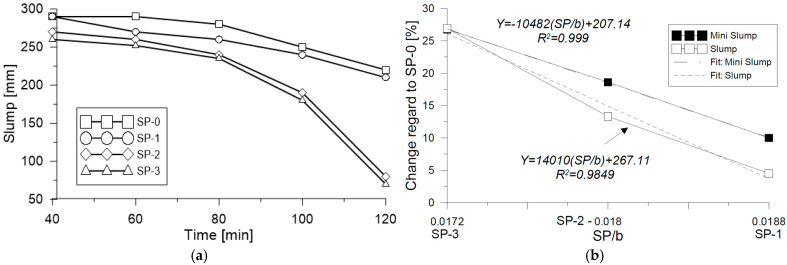
Comparison of (**a**) fluidity and (**b**) laying through laboratory tests.

Fresh state mix fluidity behavior that was evaluated at different times (t_40, t_60, t_80, t_100, and t_120) with the mini-Slump test presented a reduction in fluidity of 28% for the SP-0 mix, which started with a Df/Do ratio (Df final diameter; D*_o_* reference diameter = 100 mm) for t_120_ (Figure 7a). For mixes SP-1, SP-2, and SP-3, there was a registered reduction in fluidity against time of 19%, 35%, and 28%, respectively. Upon comparing change in average fluidity in the range of time measured versus the superplasticizer-binder ratio (*SP/b*) in relation to the reference mix SP-0 measured with mini-Slump, an average reduction of 10.0% was identified for the SP-1 mix, 18.6% for the SP-2 mix, and 26.8% for the SP-3 mix.

**Figure 8:**
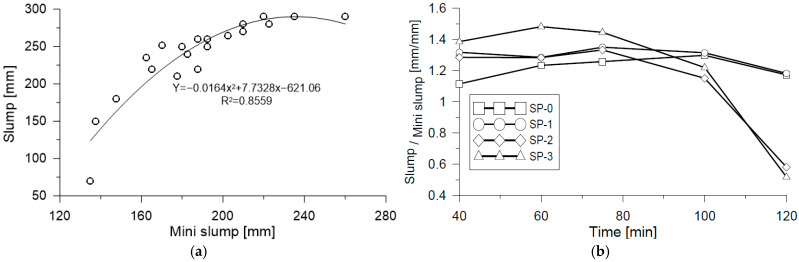
Comparison of (**a**) Slump–Mini-slump correlation and (**b**) Slump-to-Mini-slump ratio as a function of mixing time.

Likewise, it was possible to observe a linear behavior, as evidenced in Figure 7b. In conclusion, the mini-Slump test allowed us to measure the fluidity values that were identified in the mix behavior, including the high fluidity mixes evaluated in this study. On the contrary, upon conducting the same analysis of results obtained with the Slump test, it was possible to observe that the percentage of change is similar only for the mix that has a lower proportion of SP (SP-3), whereas for the SP-1 and SP-2 mixes, change is lower according to the data obtained with the mini-Slump test. These results confirm that the Slump test alone does not capture the full range of fluidity in UHPFRC mixes, as also noted in previous studies [2,3,4]. The correlations shown in Figure 8 reinforce this conclusion. As shown in Figure 8a, a strong correlation is observed between the Slump and mini-Slump tests, with a coefficient of determination of R^2^ = 0.855. In contrast, Figure 8b does not exhibit an explicit correlation value, making it difficult to quantitatively assess the relationship without further statistical analysis. These results indicate that although both tests are intended to evaluate workability, the mini-Slump test appears to be more sensitive to variations in superplasticizer content compared to the conventional Slump test.

### 3.2. Static Fluency Effort Behavior

**Figure 9:**
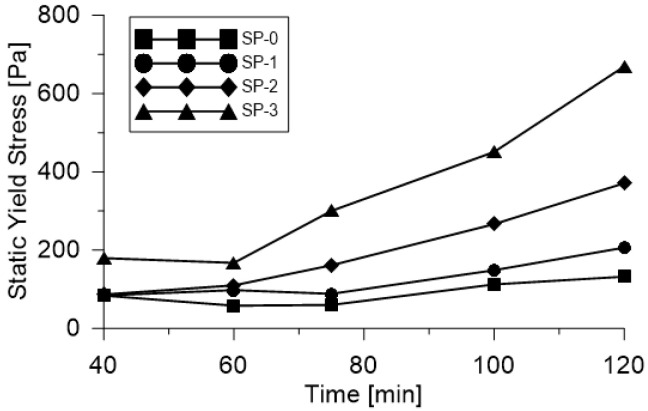
Variation of static fluency effort in time for each of the evaluated mixes.

Figure 9 illustrates the evolution of static yield stress ($\tau_{s}$) over time for the four UHPFRC mixes under study. Mixes SP-0 and SP-1, which contain the highest levels of superplasticizer, exhibited similar behavior with only a slight increase in $\tau_{s}$ observed between the measurement times t_40 and t_60. In contrast, SP-2 and SP-3, the mixes with reduced superplasticizer content, showed a pronounced increase in yield stress, particularly for times exceeding 60 min. This trend is primarily attributed to the cement hydration process, which reduces the fluidity of concrete as a function of time and temperature. This reduction in fluidity is strongly influenced by the rate of cement hydration, which accelerates with increasing temperature. As demonstrated by calorimetric analysis, the evolution of rheological properties over time is closely linked to the heat released during hydration [46]. In the fresh state, yield stress arises from the interparticle Van der Waals forces, electrostatic interactions, and the formation of a flocculated structure due to early hydration products such as ettringite and alumina gel. As time progresses, the microstructure of the paste begins to recover, and cement hydration continues at a slower rate, especially in the presence of set retarders. Eventually, the material enters a stage of accelerated hydration, during which elastic yield stress increases significantly. This is associated with the transformation of tricalcium aluminate (C₃A) compounds from a liquid to a solid phase, which contributes to a substantial increase in static yield stress [47]. In addition, the rheology of mixes containing superplasticizers is influenced by the concentration of residual polymer within the pore solution. Changes in the composition of the aqueous phase directly affect fluidity as the availability of free water for particle dispersion decreases over time [48]. Numerous studies [4,7,49,50,51,52] have established correlations between rheological parameters, obtained using the Bingham or modified Bingham models, and results from conventional workability tests such as the Slump and mini-Slump tests. Furthermore, predictive models have been proposed to link rheological measurements with empirical indicators of flowability, one of which is presented in Equation (8).

(8) $$\tau_{s}=(225\cdot \rho \cdot g\cdot V\_C^{2})/(128\cdot \pi^{2}\cdot D_{f}^{5})$$

where g corresponds to gravity acceleration, ρ is mix density, V_C is test cone volume, and $D_{f}$ is the measured flow diameter.

**Figure 10:**
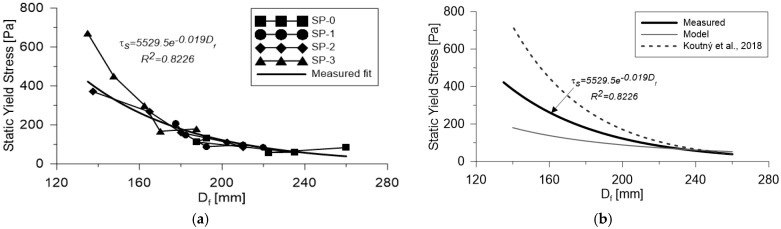
Measuring of mix fluidity. (**a**) Mini-Slump test. (**b**) Laying measurement using the Abrams cone (included a curve proposed by [7]).

By correlating the static yield stress ($\tau_{s}$) values with the flow diameter ($D_{f}$) obtained from the mini-Slump test for each of the mixes analyzed in this study, a strong relationship was observed, with a coefficient of determination ($R^{2}$) of 0.8226 (Figure 10a). The largest $D_{f}$ values correspond to mixes SP-0 and SP-1, which exhibited the highest fluidity due to their greater superplasticizer content. Conversely, the lowest flow diameters—below 180 mm—were recorded for SP-2 and SP-3, indicating reduced workability in mixes with decreased admixture dosages.

Upon applying the predictive model described in Equation (8), it was found that the experimental data align well with the model in the flow diameter range of 210 mm to 260 mm, which corresponds to the more fluid mixes. However, for less fluid mixes, the model’s predictive capacity was notably reduced as it failed to adequately fit the data—an effect clearly illustrated in Figure 10b. This discrepancy is attributed to the exponential nature of the model, which better reflects gravity-driven spreading behavior, and may not fully capture the nonlinear response of highly cohesive and thixotropic systems. Additionally, Figure 10b includes reference data from Koutný et al. [7], whose research aimed to estimate rheological parameters based on conventional laboratory and field tests, including the mini-Slump test. Their findings reinforce the observed relationship between flow diameter and yield stress for high-fluidity mixtures while also highlighting the limitations of such models when applied to low-fluidity concretes. It is also important to note that this mismatch may be influenced by the physical characteristics of the materials used. The high content of fine reactive particles (such as silica fume and calcium carbonate), together with the significant volume of steel fibers, promotes internal structural build-up and particle bridging, which intensifies the thixotropic behavior of the mix. These effects become more pronounced at lower superplasticizer dosages, where dispersion is reduced, leading to increased internal friction and reduced deformability.

**Figure 11:**
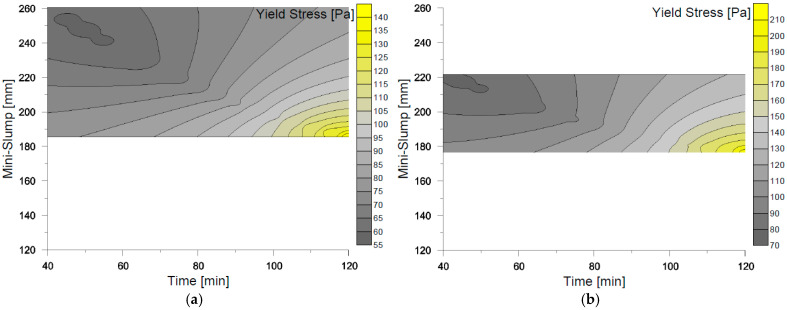

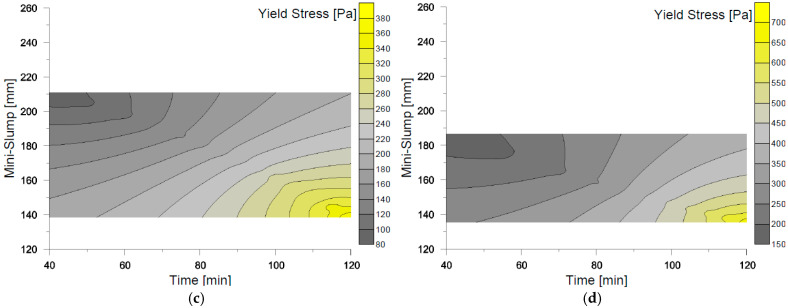
Correlation between static fluency effort and mini-Slump tests. (**a**) SP-0; (**b**) SP-1; (**c**) SP-2; (**d**) SP-3.

The results illustrating the correlation between mini-Slump test flow diameters and yield stress ($\tau_{s}$) measured at different time intervals for each of the evaluated mixes are presented in Figure 11 in the form of a contour map. This visualization highlights the evolution of rheological behavior over time, emphasizing both the variation in measurement ranges across the four mixes and the progressive increase in yield stress. Notably, for all mixes, a marked increase in $\tau_{s}$ values was observed at t_120, corresponding to a significant reduction in fluidity. This behavior is visually represented in Figure 11a–d, where each mix exhibits a distinct trajectory in the relationship between flow diameter and static yield stress over time. The contour maps clearly demonstrate that the loss of workability is more pronounced in mixes with lower superplasticizer content, aligning with the previously discussed hydration kinetics and structural build-up phenomena.

**Figure 12:**
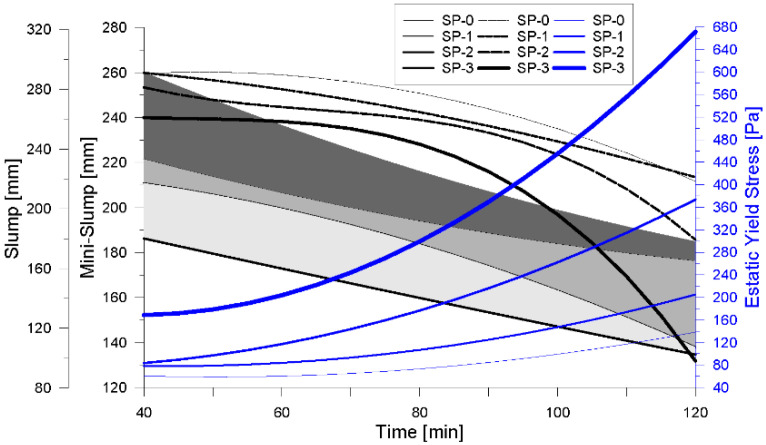
Correlation between flow tests (Slump and mini-Slump) with static flow effort in studied mixes.

The graphical model presented in Figure 12 enables the identification of static yield stress using either of the two conventional tests employed in this study, incorporating time as a critical variable that governs the workability of UHPFRC mixes. This time-based approach is especially relevant for informing construction processes such as transportation, casting, and finishing, where controlling the fresh-state behavior of the material is essential. Moreover, the ability to estimate yield stress through simple field tests enhances its practical applicability across various UHPFRC-based interventions. In particular, for pavement rehabilitation overlays, determining a critical yield stress threshold—defined in relation to the material’s intrinsic rheological properties and the geometric conditions of installation—can guide the execution of placement strategies. This concept aligns with the methodology proposed by Shin et al. [25], who emphasize the integration of rheological control into the design and construction of UHPFRC overlays.

### 3.3. Behavior of Dynamic Fluency Effort, Plastic Viscosity, and Thixotropy

**Figure 13:**
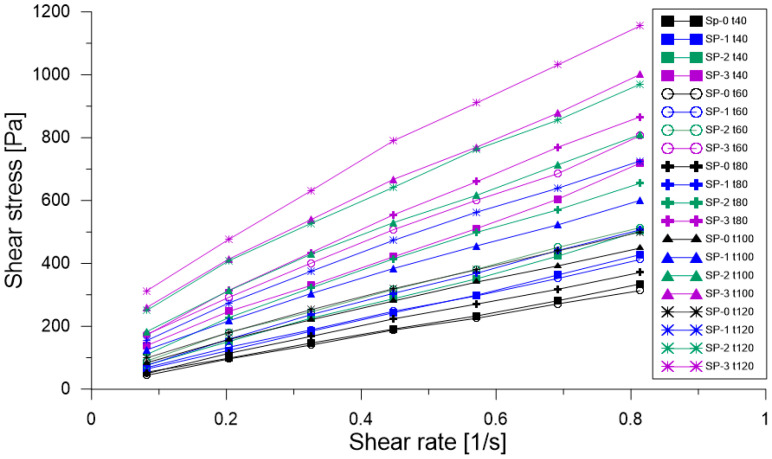
Shear effort versus shear deformation for each of the evaluated mixes.

The application of the modified Bingham model to the four concrete mixes analyzed in this study is shown in Figure 13. Across the different families of rheological curves—classified by mix type and evaluation time—a non-linear behavior was observed, indicating deviations from ideal Bingham fluid behavior.

In particular, mix SP-0 exhibited a dynamic yield stress close to zero, and in some instances, when fitted using the conventional Bingham model, it produced negative yield stress values, reflecting its high fluidity and low resistance to flow initiation. This behavior is characteristic of highly workable materials with minimal structural build-up at rest. For viscoelastic materials, flow commences only after surpassing a specific elastic limit ($\tau_{0}$); once this threshold is exceeded, a linear relationship emerges between shear stress (τ) and shear rate ($\overset{\cdot }{\gamma }$). The slope of this linear region defines the plastic viscosity ($\mu_{p}$), which quantifies the material’s resistance to continuous deformation under shear [35].

**Figure 14:**
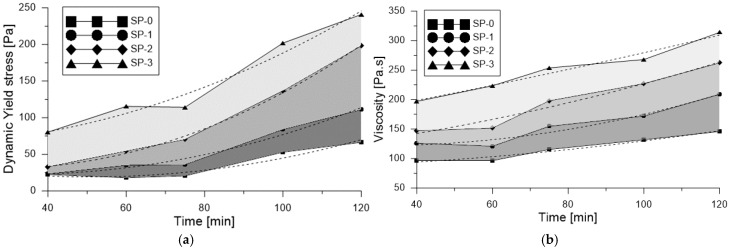
Measuring of rheological parameters, (**a**) Effect of superplasticizer in dynamic fluency effort; (**b**) Effects of superplasticizer in viscosity.

**Figure 15:**
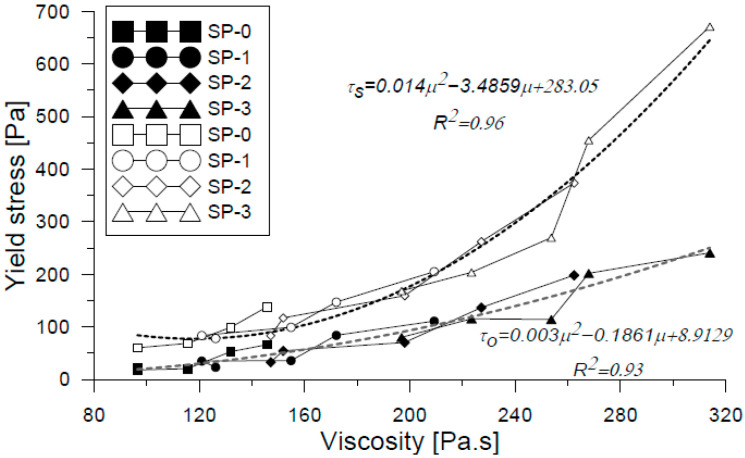
Correlation of viscosity and static and dynamic fluency effort for studied mixes.

The effect of varying superplasticizer content on dynamic yield stress for each of the studied mixes and measurement times is presented in Figure 14a, exhibiting a trend similar to that observed in Figure 9 for static yield stress. The effect of varying superplasticizer content on dynamic yield stress for each of the studied mixes and measurement times is presented in Figure 14a, exhibiting a trend like that observed in Figure 9 for static yield stress. Figure 15 provides a direct comparison between static yield stress ($\tau_{s}$) and dynamic yield stress ($\tau_{0}$) for the various studied mixes across the different measurement times. As shown, static yield stress was consistently higher than dynamic yield stress, confirming the trend reported in previous studies [35,47]. This difference is attributed to the structural nature of the material: while dynamic yield stress reflects the stress required to maintain flow in a moving mixture, static yield stress corresponds to the stress necessary to initiate flow from rest, when the internal structure has had time to rebuild. Notably, the gap be-tween $\tau_{s}$ and $\tau_{0}$ tends to increase with resting time, indicating a progressive structural build-up in the mixtures. This behavior aligns with the thixotropic characteristics commonly observed in cementitious systems modified with superplasticizers. Furthermore, the superplasticizer content significantly influences this evolution: higher dosages result in a smaller relative difference between $\tau_{s}$ and $\tau_{0}$, suggesting a reduced structuration rate, likely due to enhanced particle dispersion and the suppression of flocculated interactions. Therefore, Figure 15 not only confirms the consistent dominance of static over dynamic yield stress across all conditions but also highlights the key role of resting time and superplasticizer dosage in the structural evolution of the system.

Figure 14b shows the corresponding variation in plastic viscosity ($\mu_{p}$) over time and between mixes. Viscosity was observed to increase with both prolonged mixing time and decreasing superplasticizer dosage, in line with findings from other research [48,53,54]. One of the key factors influencing viscosity in UHPFRC is the combined effect of superplasticizer and water content, which in turn may affect particle packing density, fiber distribution, and consequently, the flexural performance of UHPFRC composites [48,54,55]. According to Riu Wang et al. [48], maintaining moderate rheological parameters is critical to ensure a uniform distribution of steel fibers in UHPFRC. Excessively high values of yield stress and viscosity can hinder fiber dispersion during mixing, while overly low values may result in segregation. For UHPFRC mixes with 1%, 2%, and 3% fiber content, optimal static yield stress values have been reported in the ranges of 900–1000 Pa, 700–900 Pa, and 400–800 Pa, respectively. When comparing these thresholds to the results of the present study, similar static yield stress values were found; however, viscosity increased substantially in mixes SP-2 and SP-3, particularly at later ages. As noted by Khayat et al. [52], UHPFRC mixes typically exhibit plastic viscosities ranging from 20 Pa·s to 200 Pa·s, and higher viscosity levels in self-compacting UHPFRC are often associated with improved fiber orientation and mechanical performance. This trend aligns with the behavior observed in SP-1 and SP-2 mixes in terms of viscosity, yet it also highlights a gap in current understanding regarding the specific influence of rheological parameters on fiber distribution across different UHPFRC formulations.

**Figure 16:**
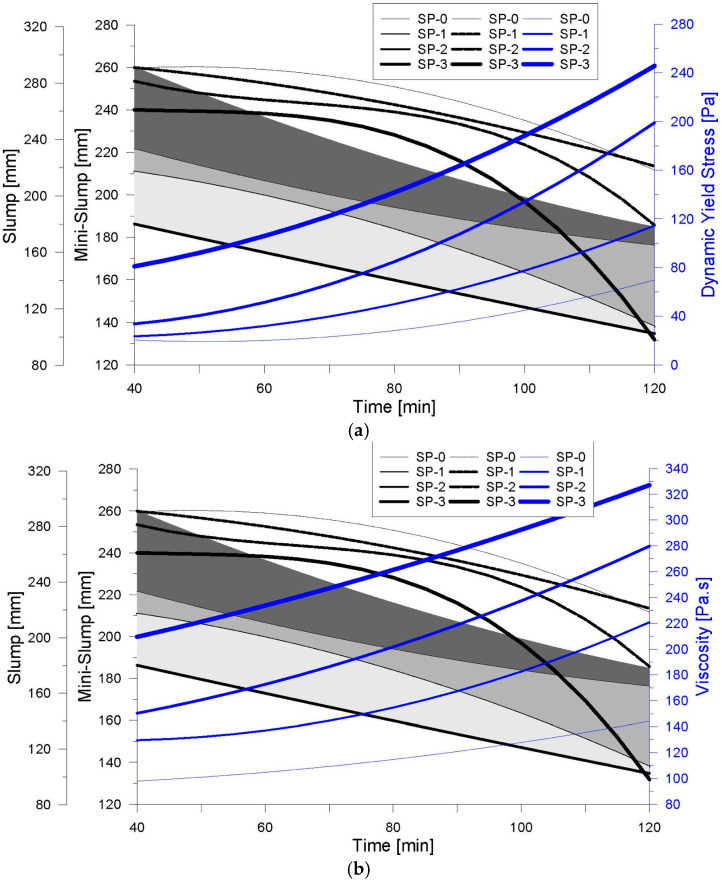
Measurement of rheological parameters. (**a**) Effects of superplasticizer in dynamic fluency effort; (**b**) effects of superplasticizer in viscosity.

**Figure 17:**
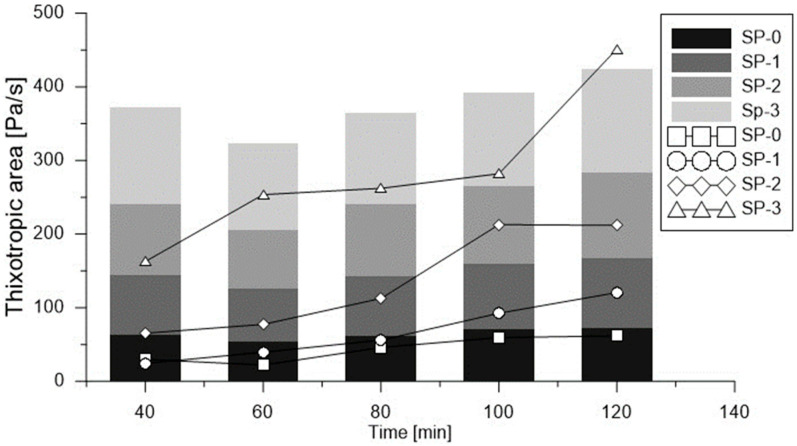
Effects of superplasticizer variation on thixotropy.

Figure 16a illustrates the relationship between dynamic yield stress and results from conventional flow tests, in a manner analogous to that previously shown for static yield stress. Additionally, Figure 16b depicts the behavior of plastic viscosity across the range of evaluated mixes, reinforcing the link between viscosity evolution and mix composition. Beyond these rheological variations, this study also included a quantitative assessment of thixotropy. As shown earlier in Figure 9, the time-dependent development of static yield stress in each mix indicates progressive changes in the material’s thixotropic behavior [56]. The results derived from the two thixotropy evaluation methods used in this study are presented in Figure 17, where noticeable differences were observed between the reported values. According to the method proposed by Ahari et al. [39], the calculated thixotropy values were higher than those obtained from the area enclosed by the ascending and descending flow curves. Nonetheless, both methods confirmed that thixotropy increased over time for all mixes. In particular, the reference mix (SP-0) exhibited the lowest thixotropy values, yet when the change in thixotropy between t_40 and t_120 was analyzed, all mixes showed an average increase by a factor of 3.25, indicating significant structural build-up over time. Similar values have been reported in the literature for cementitious materials [47]. Ultimately, the assessment of thixotropy proved to be a key indicator of evolving rheological properties [57], with direct implications for formwork pressure management [52,58] and material stability at rest [59,60]. Moreover, when a material remains at rest for prolonged periods during the placement process, thixotropy helps to explain the observed loss of workability [57].

### 3.4. Behavior of Mixes in Hardened State

**Figure 18:**
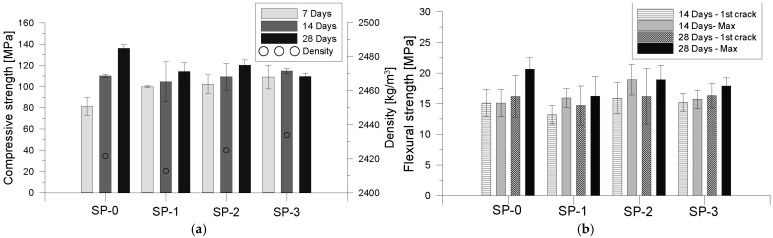
Effects of variation of superplasticizer proportion on mechanical properties of mixes. (**a**) Compressive resistance; (**b**) flexural resistance, effort at first cracking and maximum effort.

The results of the mechanical performance in the hardened state for the evaluated mixes are presented in Figure 18 [61]. Figure 18a illustrates the evolution of compressive strength at 7, 14, and 28 days for each mix. The most significant variation was observed at early ages, a trend consistent across all tested mixes. The maximum compressive strength was recorded at 137 MPa on day 28 for the reference mix (SP-0). When comparing the other mixes to the reference at the same age, an average reduction of 15.7% in compressive strength was identified. A similar trend was observed in the flexural strength results shown in Figure 18b, where mixes with lower superplasticizer content demonstrated reduced performance. These changes in mechanical behavior are closely related to workability differences among the mixes. Specifically, increased viscosity resulting from reduced superplasticizer content negatively impacted particle packing and mix homogeneity, which in turn led to lower mechanical strength. This highlights the strong interdependence between fresh-state rheology and mechanical performance in UHPFRC systems.

### 3.5. Verification of UHPFRC Mix in Installing as Pavement Overlay

Based on the mechanical performance results, the SP-2 mix was selected for application as an overlay as it exhibited both compressive strength and flexural strength values exceeding 120 MPa and 15 MPa at 28 days, respectively. In addition to its favorable mechanical properties, SP-2 also demonstrated distinct characteristics of a thixotropic mix [15], making it suitable for use in sloped or form-sensitive applications. The critical yield stress required for installation was calculated using Equation (7), and the corresponding results are summarized in Table 4, providing a foundation for evaluating the feasibility of this mix in practical pavement rehabilitation scenarios.

**Figure 19:**
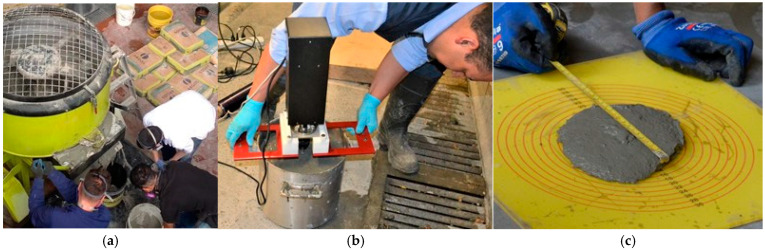
Manufacturing and control of SP-2 mix. (**a**) Mixing process; (**b**) ICAR vane rheometer; (**c**) mini-Slump test.

**Figure 20:**
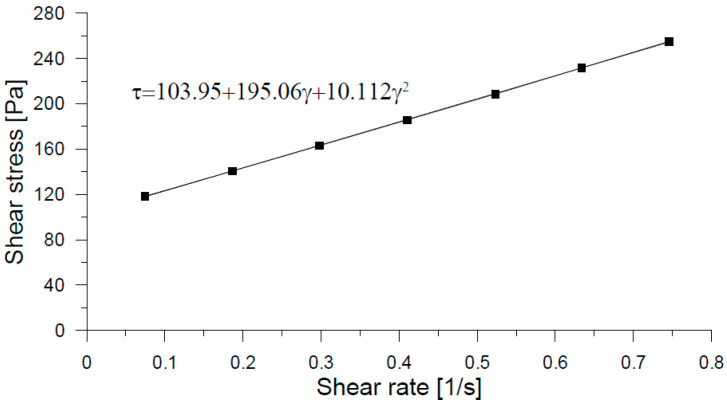
Measurement of rheological parameters for the SP-2 mix in order to be used as overlay.

Figure 19a illustrates the mixing process for the SP-2 formulation, while Figure 19b shows the corresponding rheological control test conducted using an ICAR vane rheometer, and Figure 19c presents the mini-Slump test results recorded at t_100. The mixing sequence followed the procedure detailed in Table 3 and was designed to produce a UHPFRC overlay with a target thickness of 40 mm. The measurement of rheological parameters yielded a torque value of 1.14 N·m, corresponding to a static yield stress ($\tau_{s}$) of 265 Pa. The rheological behavior of the SP-2 mix, analyzed with the modified Bingham model, is shown in Figure 20, with a calculated dynamic yield stress ($\tau_{0}$) of 103.95 Pa and a plastic viscosity ($\mu_{p}$) of 195.06 Pa·s. Additionally, the average mini-Slump flow diameter measured at t_100 was 165 mm, as indicated in Figure 19c, confirming the mix’s moderate workability and compatibility with thixotropic overlay applications.

**Figure 21:**
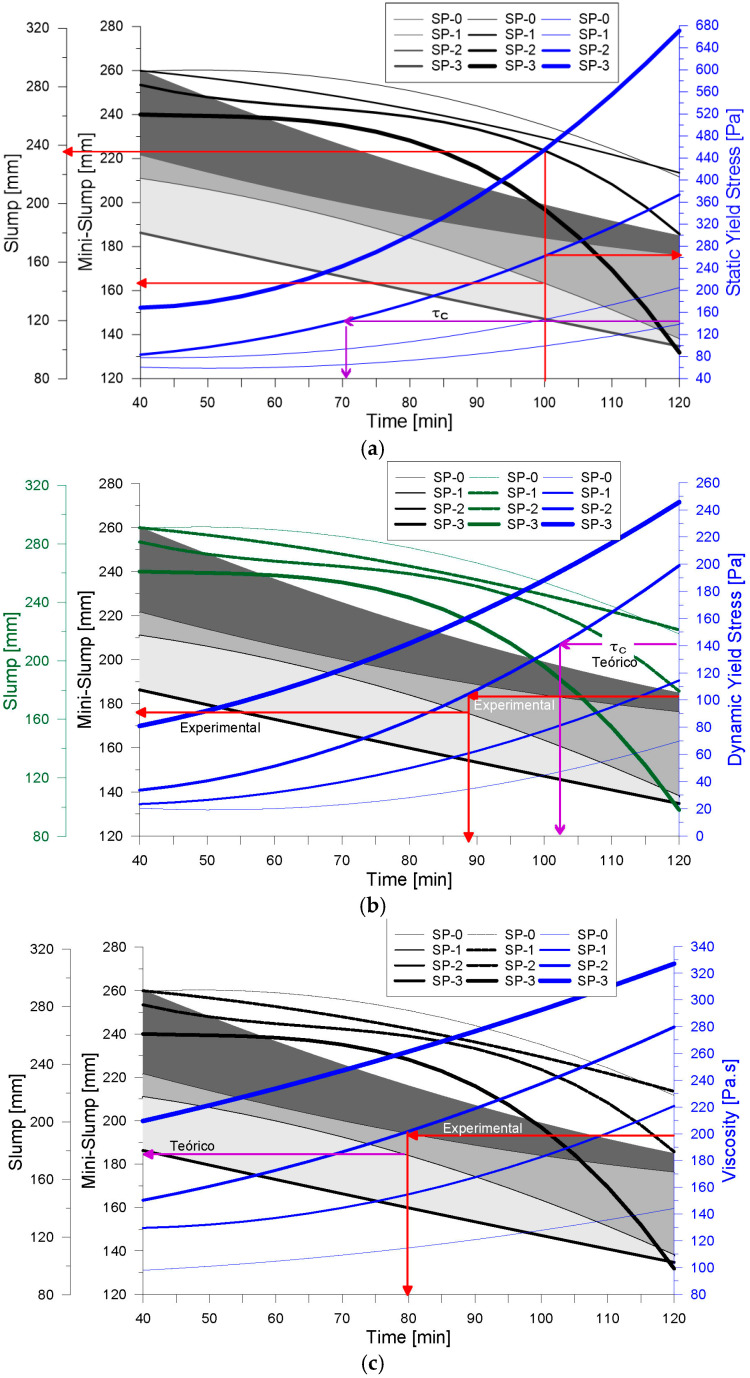
Correlation of rheological parameters and fluency tests. (**a**) Slump, Mini-Slump and Elastic Yield Stress vs. Time. (**b**) Slump, Mini-Slump and Dynamic Yield Stress vs. Time. (**c**) Slump, Mini-Slump and Plastic Viscosity vs. Time.

**Figure 22:**
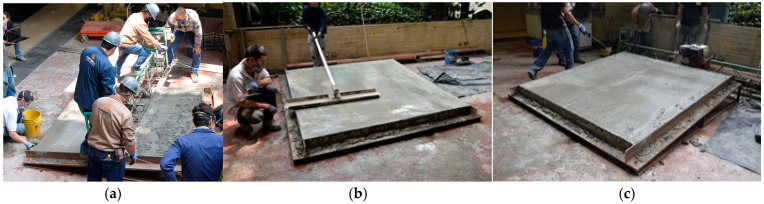
Installing of the UHPFRC overlay with 40 mm in thickness. (**a**) Placement of the UHPFRC mix over the prepared formwork under field conditions; (**b**) leveling process of the overlay to ensure uniform 40 mm thickness; (**c**) finalized UHPFRC overlay surface after placement, prior to curing phase.

The rheological parameters obtained from the experimental tests were immediately compared with the theoretical critical yield stress ($\tau_{c}$), as shown in Figure 21a. In this case, the measured static yield stress ($\tau_{s}$) exceeded the critical value, indicating that the concrete would not flow under gravity on the inclined slab, a behavior consistent with findings reported in previous studies [25,42]. Furthermore, the mini-Slump test result was found to be in good agreement with the theoretical prediction, as demonstrated by the correlation of parameters. Following this verification, the installation of the overlay was carried out under field conditions, as documented in Figure 22, confirming the mix’s suitability for application in thixotropic overlay systems on sloped surfaces.

Although the mix control process in this case was primarily based on the comparison between the static yield stress ($\tau_{s}$) and the critical yield stress ($\tau_{c}$), as well as the flow diameter obtained from the mini-Slump test, additional parameters such as dynamic yield stress ($\tau_{0}$) and plastic viscosity ($\mu_{p}$) were also evaluated. As shown in Figure 21b, the experimentally determined $\tau_{0}$ value of 103 Pa, when plotted on the response graph for the SP-2 mix, corresponds to an estimated mixing time of 88 min and a flow diameter of 176 mm. This value may, therefore, be defined as the critical dynamic yield stress ($\tau_{cd}$). Furthermore, the analysis of viscosity behavior in Figure 21c reveals that the associated time for placement is reduced in comparison to the control criteria presented in Figure 21a. This reduction suggests improved workability of the mix during the placement phase, particularly when extending the material over the pavement surface. As discussed by Tae Yong Shin et al. [25], increased fluidity facilitates filling capacity, supporting the conclusion that low-viscosity mixes allow for faster filling, which minimizes the presence of voids within the structure. However, in inclined surfaces or ramp applications, mixes with higher viscosity and higher yield stress are recommended to ensure stability and prevent uncontrolled flow during placement.

### 3.6. Practical Implications, Limitations, and Future Directions

Practical implications of this research are based on the rheological adjustment of Ultra-High-Performance Fiber-Reinforced Concrete (UHPFRC) mixes specifically designed for pavement overlays on inclined surfaces. While UHPC is typically designed to maximize flowability for self-compacting applications, this study addresses the opposite challenge: developing a thixotropic mix with sufficient static yield stress to resist gravitational flow on slopes, without compromising its characteristic ultra-high flexural performance.

An important contribution is the minimalistic approach to modifying the reference UHPFRC mix adjusting only the superplasticizer to binder ratio (SP/b) while keeping the rest of the formulation constant. This strategy provides a systematic and practical framework to shift the rheological profile toward increased yield stress and viscosity, enabling the control of structural build-up over time.

Additionally, the study proposes a predictive analytical model for critical yield stress adjustment to thin overlays on sloped pavements, correlating rheological parameters with conventional workability tests (mini-Slump and Abrams cone) and demonstrating high predictive capacity within specific flow ranges.

Unlike prior research limited to laboratory scale validation, this work includes a real-scale application of the selected UHPFRC mix (SP-2), installed as a 40 mm overlay on a 10% slope. Importantly, this trial was conducted on two typical base materials used in pavement rehabilitation, conventional concrete, and asphalt layer. These materials introduced realistic surface textures and friction conditions, reinforcing the practical viability of the proposed formulation for field implementation.

This integrated rheological, mechanical, and field-validation approach positions the study as a significant contribution to the advancement of UHPC technology in infrastructure rehabilitation, particularly for slope-sensitive pavement applications.

While the results presented demonstrate the effectiveness of the rheological adjustment strategy and its feasibility for field application, the study also presents certain limitations that should be acknowledged for future research:

- Restricted range of rheological modifications: The experimental program focused exclusively on varying the superplasticizer-to-binder ratio (SP/b), without exploring the combined effects of other parameters such as water content, fiber volume fraction, particle size distribution, or alternative admixtures. As a result, the findings are constrained to a narrow formulation window and may not be directly extrapolated to other UHPFRC systems.
- Constant fiber content across mixes: The steel fiber content was held constant in all formulations, which limited the analysis of how varying fiber volume may influence rheological behavior or mechanical performance. As fiber content can affect viscosity, dispersion, and flexural behavior, future studies should evaluate the combined effects of fiber dosage and rheology, particularly under thixotropic conditions.
- Single slope configuration and fixed overlay thickness: The field application was performed on a 10% slope with a 40 mm overlay thickness, limiting the validation of the predictive model for critical yield stress to this specific geometry. Different slope angles, thicknesses, or multilayer configurations could introduce additional flow or stability phenomena not captured in the current setup.
- No monitoring under service loads or environmental exposure: Although the field trial was carried out on realistic base layers (asphalt and concrete), it was not subjected to mechanical loading or environmental cycles.
- Recent efforts to model complex mechanical behavior in concrete using machine learning and deep learning approaches [62] suggest a growing interest in data-driven prediction, which could complement experimental investigations such as the present study.

These limitations define the scope of the study and offer opportunities for further development, particularly in extending the applicability of the proposed methodology to a broader set of field conditions and design scenarios.

## 4. Conclusions

This study assessed the impact of modifying the superplasticizer content in a self-compacting UHPFRC reference mix, focusing on both fresh-state rheological properties and hardened-state mechanical performance, with the aim of validating the use of a thixotropic UHPFRC for overlay construction in pavement rehabilitation, specifically on a 10% slope. Based on the experimental program, the following conclusions were drawn:

This study demonstrated that adjusting the superplasticizer-to-binder (SP/b) ratio has a significant impact on the rheological behavior of Ultra-High-Performance Fiber-Reinforced Concrete (UHPFRC) mixes intended for pavement rehabilitation overlays on sloped surfaces. Reductions of 4%, 8%, and 12% in SP content relative to the reference mix resulted in measurable increases in static and dynamic yield stress, as well as thixotropic build-up, without compromising the characteristic ultra-high flexural performance of UHPFRC.

A strong correlation was identified between conventional flow tests (e.g., mini-Slump) and rheological parameters, supported by regression models that allowed for the prediction and validation of UHPFRC behavior. These findings confirm the potential for using conventional tests alongside rheological analysis to evaluate and control workability in UHPFRC systems. Moreover, the study captured the time-dependent evolution of mix properties, contributing to the understanding of structural build-up and thixotropic effects.

Additionally, real-scale application trials were conducted on two typical rehabilitation substrates: concrete and asphalt. The overlay was installed on a 10% longitudinal slope using conventional screeding techniques. The adjusted mixes—especially the 8% reduction variant—demonstrated excellent surface stability and shape retention immediately after placement, with no evidence of gravitational flow, deformation, or segregation. These results confirm the practical feasibility of the proposed rheological tuning approach under critical slope and friction conditions.

Future work should focus on evaluating the long-term durability and fatigue performance of these mixes under traffic and environmental loads, as well as investigating the role of fiber content and fiber–matrix interaction in the rheological and mechanical behavior of UHPFRC systems.

## Figures and Tables

**Table 1:** Physical properties of superplasticizer (SP).

Superplasticizer (SP)	Units	Physical Properties
Color	-	Amber
Specific density	kg/m^3^	1136.0
pH, 20 °C	-	4.7
Marsh Viscosity	mPa.s	114

**Table 2:** Mix proportions (in kg/m^3^) for the different UHPFRCs.

Material/Mix	SP-0	SP-1	SP-2	SP-3
Portland cement	740	740	740	740
Calcium carbonate	310	310	310	310
Silica fume	210	210	210	210
Steel fiber	153	153	153	153
Siliceous sand	802	802	802	802
Effective water	219.0	219.8	220.5	221.2
Superplasticizer (SP)	24.7	23.7	22.7	21.7
SP/b	0.0196	0.0188	0.0180	0.0172

**Table 3:** Details of the mixing procedures, testing, and sampling.

Step	Start Time	Finish Time	Description of Action
	(mm:ss)	(mm:ss)	
1	0:00	1:00	Mix SP + Water
2 = (t_0)	0:00	0:30	Add 50% dry materials
3	0:30	1:30	Mix
4	1:30	2:00	Add 25% dry materials
5	2:00	8:00	Mix
6	8:00	8:30	Add 25% dry materials
7	8:30	27:00:00	Mix
8	27:00:00	27:30:00	Add fiber
9 = (t_f)	27:30:00	31:00:00	Mix
10	31:00:00	37:00:00	Mix
11	37:00:00	40:00:00	Test preparation
12 = (t_40)	40:00:00	48:00:00	Simultaneous Tests
13 = (t_60)	60:00:00	68:00:00	Simultaneous Tests ^a^
14 = (t_80)	80:00:00	88:00:00	Simultaneous Tests ^a^
15=(t_100)	100:00:00	108:00:00	Simultaneous Tests ^a^
16 = (t_120)	120:00:00	128:00:00	Simultaneous Tests ^a^

^a^ Steps 10 and 11 were repeated to later started with the simultaneous tests.

**Table 4:** Measuring of critical static fluency effort.

Parameter λ		0.84
Density ρ	$kg/m^{3}$	2459.3
Gravity g	$m/s^{2}$	9.81
Thickness d	m	0.040
Slope in percentage	%	10
$\tau_{c}$	Pa	142.7

## Data Availability

The data presented in this study are available on request from the corresponding author (UHPC mix developed by researchers from Pontificia Universidad Javeriana) due to legal and intellectual property restrictions. The formulation, materials, and procedures are proprietary and protected as research assets of the authors and their institutions.
